# Multi-venous thrombosis associated with a perforated hydatid cyst: A rare case report and comprehensive literature review

**DOI:** 10.1016/j.idcr.2025.e02297

**Published:** 2025-06-20

**Authors:** Lotfollah Davoodi, Eissa Soleymani, Pooria Sobhanian, Mahla Hosseinzadeh, Mansoureh Baradaran, Elham Sadat Banimostafavi, Mahdi Fakhar

**Affiliations:** a Department of Infectious Diseases, Antimicrobial Resistance Research Center, Communicable Diseases Institute, Mazandaran University of Medical Sciences, Iran; b Department of Parasitology, Toxoplasmosis Research Center, Communicable Diseases Institute, Mazandaran University of Medical Sciences, Mazandaran, Sari, Iran; c Student Research Committee, Faculty of Medicine, Mazandaran University of Medical Sciences, Sari, Iran; d Department of Radiology, Imam Ali Hospital, North Khorasan University of Medical Science, Bojnurd, Iran; e Department of Radiology, Shahid Beheshti Hospital, Qom University of Medical Sciences, Qom, Iran; f Iranian National Registry Center for Lophomoniasis and Toxoplasmosis, Imam Khomeini Hospital, Mazandaran University of Medical Sciences, Sari, Iran; g Department of Medical Microbiology and Immunology, School of Medicine, Qom University of Medical Sciences, Qom, Iran

**Keywords:** Albendazole, *Echinococcus granulosus*, Hydatid cyst disease, Venous thrombosis, Warfarin

## Abstract

**Background:**

Hydatid cyst disease (HCD), caused by the larvae of *Echinococcus granulosus* (*E. granulosus*), can lead to rare vascular complications. This case report details the diagnosis and management of a patient with a hepatic hydatid cyst complicated by extensive multi-venous thrombosis.

**Case presentation:**

A 61-year-old male stockbreeder presented with abdominal pain localized in the hypogastric region, exacerbated by food intake. Imaging revealed a hepatic HCD and thrombosis of the portal, superior mesenteric, and splenic veins. The patient was successfully treated with albendazole for the cyst and anticoagulant therapy for the thrombosis, with no recurrence of symptoms during a three-year follow-up period.

**Conclusion:**

While multi-venous thrombosis associated with HCD is rare, documented cases highlight the need for increased awareness among physicians to effectively recognize and manage this complication.

## Introduction

Hydatid cyst disease (HCD) is a silent zoonotic parasitic disease caused by the larvae of a parasitic worm called *Echinococcus granulosus* (*E. granulosus*) [1], [2], which is common in Iran and many parts of the world [3], [4]. The transmission to human occurs through the consumption of food or water contaminated with feces from infected animals, such as dogs and wolves [5]. Once ingested, the parasite enters the digestive tract and releases oncospheres that penetrate the intestinal wall and migrate to various organs via the portal venous system [6], [7], [8]. HCD primarily affects the liver in 60–70 % of patients [5], [9], although other organs such as the lungs, and brain can also be involved [10], [11]. The liver involvement in HCD can manifest in two distinct forms: alveolar and cystic [12].

The cystic form is characterized by the slow growth of the cyst (1 mm to 5 mm in diameter per year), which may remain asymptomatic for a long time. However, when the cyst grows larger, it may cause symptoms such as hepatomegaly, upper abdominal quadrant pain, fever, nausea, vomiting, leukocytosis, and increased C-reactive protein (CRP) [13], [14]. The cyst even may also rupture, causing anaphylactic shock [15]. The primary approaches for managing liver cysts are surgery, medicine therapy and even occasionally systemic chemotherapy [16]. Vascular complications, such as portal vein thrombosis, are rare but carry high mortality [17], [18]. This case report details a patient who experienced thrombosis in the portal vein, superior mesenteric vein (SMV), and splenic vein due to a ruptured hepatic HCD, representing a rare and serious complication associated with this condition. Additionally, published papers on other vein involvement associated with HCD have been reviewed.

## Case presentation

A 61-year-old man stockbreeder presented to the emergency department complaining of abdominal pain localized in the hypogastric region and exacerbated by food intake. The pain, which was initially mild in onset two weeks prior, had progressed to severe over the past two days. The patient denied experiencing anorexia, fatigue, or weight loss. His medical history revealed underlying conditions of diabetes mellitus, hypertension, and benign prostatic hyperplasia, managed with regular medications including Metformin, Losartan, and Finasteride. There were no reported drug or food allergies in the patient's history.

In physical examination, abdominal distension was apparent. Tenderness in the hypogastric region and splenomegaly were detected, while there was no indication of hepatomegaly or jaundice. The presence of ascites was confirmed through the fluid test and shifting dullness, which was subsequently tapped. The cytology of ascites did not reveal any signs of malignancy and biochemistry analyzing of ascetic fluid were normal. Also, blood test demonstrated a high serum-ascites albumin gradient (SAAG). Moreover, the leukocytosis (22,600 µL) and ESR increasing (26 mm/h) detected in the laboratory tests supported the presence of an infection. The immunoassay analysis was donned using the commercial ELISA test (Pishtaz Teb, Iran). The tests for HBS Ag, HBc Ab, and HIV Ab indicated negative results; however, positive results for antibodies against *E. granulosus* were observed, which finally facilitated the diagnosis of HCD in the patient. Also, the abdominal ultrasound (US) revealed a hypoechoic solid cystic mass measuring 60 × 70 mm in the vicinity of the left lobe of the liver.

**Fig. 1:**
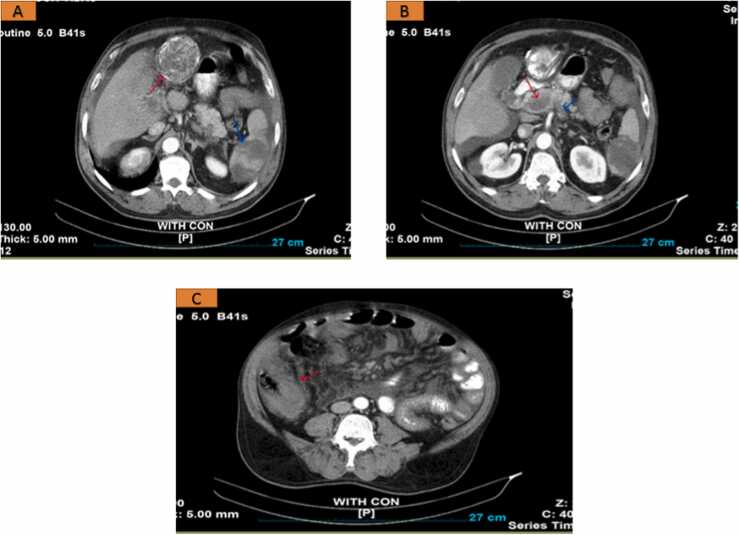
An abdominal CT scan with oral and IV contrast on a patient with portal, splenic and mesenteric venous thrombosis associated with HCD.

An abdominal computed tomography scan (CT scan) with oral and IV contrast revealed a non-enhancing heterogeneous mass measuring approximately 70 × 65 mm, with peripheral calcification in the left lobe of the liver, compatible with HCD, as indicated by the red arrow. Some hypodensities with cortical involvement were visible in the spleen, indicative of splenic infarcts (blue arrow) (Fig. 1-A). Filling defects in the delayed phase in the portal vein (red arrow) and splenic vein (blue arrow) were visible, indicating venous thrombosis (Fig. 1-B). There was also evidence of bowel wall thickening, particularly in the ileum and cecum, along with a moderate amount of ascites. The red arrow in Fig. 1-C indicates the cecum. The aforementioned findings suggest a complicated ruptured hydatid cyst accompanied by multi-venous thrombosis. Testing for Factor V Leiden mutation, protein S, and protein C resulted normal, and there was no family history of thrombosis too.

A Doppler US examination of the abdominal vessels was conducted, revealing filling defects in the right and main portal vein, SMV, and splenic vein, suggestive of portal vein and SMV thrombosis. Additionally, there was evidence of infarct and cystic changes within the spleen. Albendazole 400 chewable tablets were administered for treatment of the liver HCD. For thrombosis during his hospitalization, the patient was administered heparin injections and prescribed Warfarin in tablet form upon discharge, primarily. Following a two-month period, Apixaban was introduced, and repeatedly both PTT and PTT levels were checked. Despite the persistence of the liver cyst, there has been no recurrence of symptoms during this follow-up period. Monthly follow-up visits were conducted for six months, after which the repeatedly was done to every three months.

## Discussion

Although vascular complications of HCD are rare, factors such as a large cyst due to external compression may increase the risk of complications such as thrombosis [19], [20]. Thus,we conducted a comprehensive literature review on venous thrombosis associated HCD using the keywords “HCD” and “Venous Thrombosis” and related synonyms in Medline, Embase, Scopus, and Google Scholar (see Table 1). Twenty reported instances of venous thrombosis were found, 18 of which were associated with liver HCDs, and the remaining two patients had HCDs located in the brain and pancreas. Among the 20 reported cases in the Table 1, 11 were female and 9 were male, showing no significant disparity in the sex distribution of the affected patients to HCD and thrombocytosis, simultaneously. The mean age across these cases was 47.8 yrs, which does not closely align with the age of our patient. Additionally, 14 cases (70 %) reported abdominal pain, akin to the symptoms observed in our case. The obstruction caused by portal vein thrombosis can progress to involve intrahepatic portal branches or may extend distally to affect the splenic or mesenteric veins [21]. According to the literature, involvement of the portal vein was more prevalent than other veins such as the IVC, hepatic veins and SMV.

**Table 1:** Literature review of venous thrombosis due to HCD.

**Study**	**Author**	**Year**	**Age**	**Gender**	**Signs and symptoms**	**Location of HCD**	**Involved vein**	**Treatment**	**Follow up/Out come**
1	Gil Egea et al. [15]	1998	84	Female	Not reported	Liver	Portal vein	Albendazole	NA
2	S Gruttadauria et al. [30]	2003	21	Female	Mild, dull and persistent pain and palpable mass in the right upper quadrant,	Liver	Retrohepatic vena cava	Albendazole 400 mg twice a day, heparin sulfate (18 unit/kg/hr)	NA
3	Kayacetin et al. [40]	2004	63	Male	Fever, jaundice, epigastric mass, anorexia and abdominal discomfort	Liver	Portal vein	Parenteral ciprofloxacin and metronidazole empirically, Laparotomy, Choledochoduodenostomy and cystectomy, albendazole 400 mg postoperatively	Persistence of portal vein thrombosis
4	Spanou et al. [37]	2006	74	Male	Not reported	Liver	Portal vein	Albendazole and drainage of HCD content, performed by PAIR^1^	NA
5	Dülger et al.[41]	2007	21	Female	Malaise, weight loss, , moderate tenderness in the right upper quadrant, mild ascites, palpable liver edge, palpable splenic tip	Liver	Hepatic vein	Abdominal paracentesis, Albendazole (10 mg/kg/day) and surgical resection	NA
6	I Sakçak et al.[42]	2012	12	Female	Abdominal distention, ascites	Liver	Inferior vena cava (IVC)	Segment 2–3 liver graft, drainage of the liver hydatid, end-to-side anastomosis between hepatic vein and aortic graft	4 months/improved signs and symptoms
7	Moisan et al. [43]	2012	62	Female	abdominal pain with spontaneous resolution, painless hepatomegaly	Liver	Portal vein	Albendazole and variceal bleeding prophylaxis with propranolol	Asymptomatic
8	Khalid Rasheed et al. [39]	2013	42	Female	Right upper quadrant pain, jaundice fever, vomiting, dry mucous membranes, scleral icterus, distention of abdomen, tender hepatomegaly	Liver	IVC, right portal vein, and main portal vein	NBD^3^ in the left hepatic ductal system, albendazole for 3 months and oral warfarin for 6 months, catheter-directed thrombolytic therapy (250,000 units of streptokinase was given followed by infusion of 100,000 units/h for 36 h), .	Improving with no recurrence
9	Kirmizi et al. [1]	2016	33	Male	Abdominal distention after meals	Liver	Portal vein	Partial cystectomy, omentoplasty, meso-caval shunt and postoperative adjuvant albendazole (800 mg per day), anticoagulant therapy	Regression in symptoms and endoscopic varices in the postoperative period.
10	F Sabzi et al. [44]	2017	43	Male	Abdominal distention, lower extremity edema, severe dyspnea, tension ascites	Liver	Left hepatic vein and IVC	Open-heart surgery with removal of right atrial, IVC and pulmonary artery emboli.	NA
11	Ozsay O et al. [45]	2018	23	Female	Epigastric pain, tenderness in the epigastric region.	Pancreas	Splenic vein	Intravenous fluid and anticoagulant therapy, distal pancreatectomy, splenectomy, albendazole for 3 months at a dose of 800 mg/day	6 months/ no sign of recurrence or complications
12	Linhares et al. [29]	2018	62	Male	Abdominal pain in the right hypocondrium	Liver	Portal vein	Hepatectomy	NA
13	Berkane et al. [46]	2020	46	Male	Pain in the right hypochondriu, jaundice, discolored stools, dark urine, pruritus, scratching lesions	Liver	Portal vein	Total cystectomy and evacuation of the daughter vesicles, installation of external biliary drainage and a right subphrenic drainage, Albendazole tablet 400 mg / day	Asymptomatic
14	Marlasca et al. [47]	2021	79	Female	Epigastric and right upper quadrant abdominal pain, weight loss and abdominal distension,	Liver	Portal vein	Albendazole	After a few months the follow up was discontinued because the patient moved to anoyher city
15	Ben Hassine [48]	2021	47	Male	Abdominal pain	Liver	Portal vein	Albendazole- anticoagulant therapy	NA
16	Ammar et al. [49]	2021	70	Female	Pain in the abdominal right upper quadrant, healed right subcostal scar with an incisional hernia,	Liver	Portal vein	Right hepatectomy- evacuation of hydatid contents in portal vein, Transcystic biliary drainage	NA
17	Milani et al. [50]	2022	66	Female	Severe abdominal pain, Hepatomegaly and splenomegaly	Liver	Hepatic veins, portal vein, IVC,	Ceftriaxone and Albendazole 400 mg twice a day- Heparin infusion for 5 days overlapping with warfarin and then only warfarin	Candida for liver transplantation
18	M Kaeedi et al.[51]	2023	35	Female	Transient visual obscurations and persistent dull headaches in the occipital and bi-temporal regions, bilateral papilledema	Brain	Cerebral Venous Sinus	Heparin (1000 unit/h) (switched to warfarin (5 mg) after 10 days), albendazole (400 mg bid), brain surgery through right sub-occipital craniotomy	improved completely
19	Steven Lin et al. [52]	2024	12	Male	intermittent hematochezia and fatigue, abdominal pain and chronic constipation, diarrhea,	Liver	Portal vein	Albendazole, PAIR, splenectomy, meso-rex bypass	6 months/ All symptoms except hematochezia and cavernous transformation improved
20	A Moradi et al. [53]	2024	62	Male	Without any signs and symptoms	liver	Portal vein, superior mesenteric vein	Albendazole	A 6-month-long period/ patient's rejection of surgery
21	Our case	2025	61	Male	Abdominal pain localized in the hypogastric region	Liver	Portal vein, superior mesenteric vein, splenic vein	Albendazole Heparin, Warfarin and apixaban	3 years/ Improving the thrombosis

**PAIR**: Puncture, aspiration, injection, and reaspiration; **ERCP**: Endoscopic retrograde cholangiopancreatography; **NBD**: Naso Biliary Drain; **MRV**: Magnetic resonance venography; **DSA**: Digital subtraction angiography.

Portal vein thrombosis is a significant condition that can result in portal hypertension [22]. A notable clinical marker for portal hypertension is a high SAAG [23], which is often seen in various conditions, including Budd-Chiari syndrome (BCS) [24]. BCS is characterized by obstruction of hepatic venous outflow due to thrombosis or phlebitis affecting the hepatic veins and inferior vena cava [25]. The classic clinical presentation of BCS includes a triad of symptoms: hepatomegaly, ascites, and abdominal pain [26]. Thus, in the case study we examined, the diagnosis of Budd-Chiari syndrome was not established, which contrasts with the four cases documented in Table 1.

Ultrasonography is the primary imaging modality for the diagnosis, differential diagnosis, staging, and interventional management of HCDs [27], as evidenced in 12 reported studies in Table 1. In situations where ultrasound imaging is inadequate, such as in patients with HCDs in the brain, the presence of calcifications, or in obese patients, CT scan is recommended [28]. Linhares et al., demonstrated that CT scans can also be employed to diagnose venous thrombosis in addition to cysts [29]. While a study in 2003 combined inferior vena cava angiography with CT scans for the diagnosis of both cyst and venous thrombosis [30]. However, Doppler US stands out as the preferred method for identifying vascular thrombi [31]. Although HC are often diagnosed by CT scan and US, sometimes the diagnosis may become challenging due to the atypical appearance of the cyst [31]. In such cases, MRI can be helpful for revealing the exact location of the cyst and its relationship with adjacent structures [32]. While routine endoscopic retrograde cholangiopancreatography (ERCP) is not yet a standard practice for uncomplicated HCDs [33], [34].

Albendazole is recommended as the primary established therapy for hepatic HC [35] and has significantly contributed to the management of literature patients, including the one we managed. However, surgical intervention continues to be the “gold standard” approach in managing this condition [36]. Different surgical procedures can be performed according to the patient's clinical status and location of the cyst. Choledochoduodenostomy, partial or total cystectomy and hepatectomy are some potential options introduced in Table 1. E. Spanou et al. utilized the PAIR (puncture, aspiration, injection, and re-aspiration) technique to percutaneously drain the contents of the hepatic and peripheral pelvic HCD in their patient as a therapeutic intervention [37]. Anticoagulants represent the primary therapeutic approach for venous thrombosis [38]. Warfarin and heparin were the most frequently prescribed anticoagulants among the patient population evaluated in this study. A investigation conducted in the United States suggested that catheter-directed thrombolytic therapy may also be effective in such patients [39]. Although multi-venous thrombosis linked to HCD is an uncommon complication, the recorded cases underscore the necessity for heightened awareness among clinicians to effectively identify and manage this possible risk.

## Conclusion

Multi-venous thrombosis associated with hepatic HCD is rare but serious. Increased awareness among clinicians is essential for timely diagnosis and management. This case underscores the importance of thorough evaluation in HCD patients to mitigate thrombosis risks.

## CRediT authorship contribution statement

**Lotfollah Davoodi:** Writing – original draft, Methodology, Investigation. **Eissa Soleymani:** Writing – review & editing. **Elham Sadat Banimostafavi:** Software, Conceptualization. **Mahdi Fakhar:** Writing – review & editing, Software, Project administration, Investigation. **Mansoureh Baradaran:** Methodology, Data curation. **Mahla Hosseinzadeh:** Investigation. **Pooria Sobhanian:** Writing – original draft, Investigation, Formal analysis, Data curation.

## Ethics

All ethical principles are considered in this article. The patient has duly furnished written informed consent to disseminate this manuscript.

## Funding

None.

## Consent

We declare that appropriate written informed consent was obtained for the publication of this manuscript and associated images.

## Declaration of Competing Interest

We have no conflicts of interest to disclose. All authors declare that they have no conflicts of interest.
